# Comparing Fourteen Weeks of Multicomponent Training Versus Combined Training in Physically Inactive Older Women: A Randomized Trial

**DOI:** 10.3390/ijerph20032699

**Published:** 2023-02-02

**Authors:** Guilherme da Silva Rodrigues, Karine Pereira Rodrigues, Mariana Luciano de Almeida, Andressa Crystine da Silva Sobrinho, Natalia Yumi Noronha, Cicero Jonas Rodrigues Benjamim, Sabrina da Silva, Jhennyfer Aline Lima Rodrigues, Carlos Roberto Bueno Júnior

**Affiliations:** 1 Department of Internal Medicine, Ribeirão Preto Medical School, University of São Paulo, Ribeirão Preto 14040-907, SP, Brazil; 2 Ribeirão Preto College of Nursing, University of São Paulo, Ribeirão Preto 14040-907, SP, Brazil; 3 School of Physical Education of Ribeirão Preto, University of São Paulo, Ribeirão Preto 14040-907, SP, Brazil

**Keywords:** exercise training, physical activity, aging

## Abstract

Background: Combined (CT) and multicomponent training (MT) presents several benefits for aging individuals. However, the literature does not provide evidence on which of the two physical training models can better enhance improvements in physical capacity and health parameters in middle-aged and older women. Objective: The aim of this study was to compare the effects of MT and CT on physical capacity, cognitive, behavioral, and psychosocial assessment, and biochemical profile of physically inactive women aged between 50 and 70 years. Methods: Participants were randomized into two groups: MT (32 women, 64.2 ± 6.4 years) and CT (39 women, 61.4 ± 4.3 years). Both training sessions had a weekly volume of 180 min, for 14 weeks, with assessments at baseline and after the training period. Results: CT showed better results when compared to MT. In the four evaluation blocks, we noticed differences in the effect size (L = large, M = moderate, S = small, and T = trivial) between the groups in 26 variables in total, highlighting the CT group (L = 11, M = 5, S = 2, and T = 8) compared to the MT group (L = 8, M = 7, S = 7, and T = 4). Our findings showed group-time differences for strength variables using the maximum dynamic repetition test in upper and lower limbs and for agility. The multicomponent training showed improvement in the functional strength of the upper limbs evaluated through the elbow flexion and extension test (*p* = 0.037), and HDL (*p* = 0.022). Conclusions: Fourteen weeks of CT showed better benefits when compared to MT.

## 1. Introduction

Currently, reaching old age is a reality for the world population. Increased life expectancy and more extended population longevity can be found in developed countries, extending to low-income populations, and developing countries, such as Brazil [1,2]. 

One of the most beneficial low-cost interventions for this population is physical exercise [3], responsible for benefits that include increased muscle mass, improved glycemic control, improved lipid profile, reduced body mass, and mood changes [4]. According to the new World Health Organization guidelines, a training volume of at least 150 to 300 min of moderate-intensity aerobic physical activity, a training volume of at least 75–150 min of vigorous-intensity aerobic physical activity, or a combination of both per week is recommended for adults and seniors [5]. It is also of great importance to reduce sedentary behavior and replace it with physical activity, even at a light intensity.

Additionally, the American College of Sports Medicine (ACSM) emphasizes the importance of concurrently performing endurance, resistance, and flexibility training for this age group to maintain independence [6]. 

Combined training (CT) and multicomponent training (MT) protocols demonstrate the reach of these benefits [7]. CT is characterized by performing strength and aerobic training in the same session [8], while MT develops three or more physical capacities performed during the same session [9]. Previous studies have shown that MT increased muscle endurance levels, positively influenced agility improvement, and developed more outstanding balance in older adults [10], reducing the risk of falls, which are common in this older population [11]. CT also yielded beneficial results and improved lower-limb strength parameters and cardiovascular endurance [10,12].

Rodrigues et al. (2021) compared with CT and MT in women aged 50 and 75, physically active for 14 weeks. As a result, both training approaches showed health-related improvements, but only participants who underwent CT could increase agility and cardiorespiratory capabilities [13].

Both training modalities demonstrate potential for improvement, mainly in terms of improvements in physical abilities. However, it has not yet been demonstrated in the literature which of the two training protocols is more appropriate for the maintenance and improvement of physical abilities and biochemical, anthropometric, and quality-of-life parameters in inactive women over 50 years of age.

This study aimed to compare the effects of 14 weeks of CT and MT on physical capacity, cognitive assessment, behavioral, psychosocial, and biochemical profiles of physically inactive women aged between 50 and 70. As a hypothesis, we adopted that, due to the specificity of the training, the CT would improve in the maximum dynamic load tests while the MT would improve in the functional strength tests. Furthermore, both training approaches would tend to improve in the other variables with the stimulus being the same.

## 2. Materials and Methods

This randomized clinical trial is described in accordance with the Consolidated Standards of Reporting Trials (CONSORT). The inclusion criteria established to participate in the study were being a woman between 50 and 70 years of age, presenting a score ≤9.11 on the Modified Baecke Questionnaire for Older Adults^14^ to be classified as physically inactive [14]. Exclusion criteria included any physical or cognitive limitation preventing participants from carrying out the physical training protocols and assessments, and not presenting a medical release certificate. Therefore, the participants with more than 25% absence from sessions during the intervention period were excluded from the analysis.

**Figure 1:**
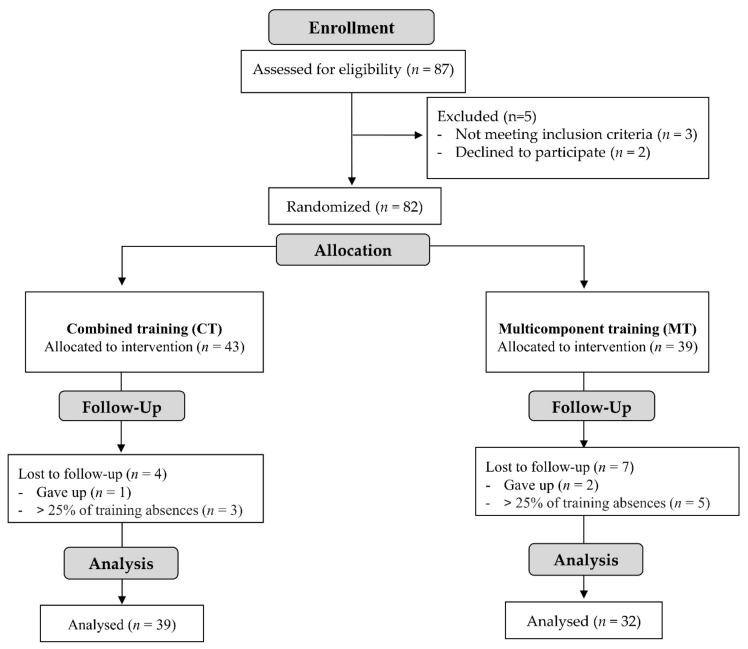
Study flowchart.

All participants signed the informed consent form approved by the ethics and research committee of the School of Physical Education and Sport of Ribeirão Preto at the University of Sao Paulo (protocol: 79582817.0.0000.5656). The signed document followed the Helsinki declaration principles. This study was also registered in the Brazilian Registry of Clinical Trials (code RBR-3g38dx full date of first registration 15 May 2018). Participants were recruited through dissemination in local media and partner social networks. In the power analysis, 32 participants per group would be needed to detect a difference between means of 3.4 kg and 21.3 kg for the primary outcomes (dynamic maximum load tests—bench press and leg press, respectively), with the alpha error probability set at 0.05 and power adjusted to 0.8. At the end of recruitment, a researcher blindly divided the participants into two groups: MT and CT (Figure 1). The randomization allocation was performed through the random number generation using Microsoft Excel software.

### 2.1. Anthropometric and Cardiovascular Measurements

The participants’ height was measured using a stadiometer (Balmak-EST-223, Brazil), and body mass was evaluated using a digital scale (G-Tech-Balgl200, G-Tech, Brazil, Ribeirão Preto). The body mass index was then calculated using the formula body mass (kg)/(height (cm^2^) [15]. Waist and hip circumference was measured using a measuring tape following the recommendations proposed by Wang et al. [14]. Blood pressure was measured using an automatic digital arm gauge, with the participant at rest for at least 5 min (SBH, 2010-OMRON^®^, model HEM-7113, Brazil) [15].

### 2.2. Functional Capacity Tests

Functional capacity was assessed using the battery of tests developed by Rikli and Jones [16], including the elbow flexion and extension tests, as well as sitting and standing up from a chair, which assessed the functional strength of the upper and lower limbs, hands on the back and sit and reach, which assessed the participants’ flexibility, and 6 min walk and dynamic agility [17], which assessed aerobic fitness and agility. In the maximum dynamic load test, participants were instructed to perform a 5 min warmup on a treadmill. Then, the specific warmup was performed. Each participant performed 20 repetitions on an inclined bench press (only with barbell) and leg press. After the 2 min break, we started the test. Participants were instructed to perform as many repetitions as possible until voluntary exhaustion, without pausing between the concentric and eccentric phases and between repetitions. The test was terminated if the participants showed exhaustion or were unable to perform the movement [12]. The maximum dynamic load test was used to assess the maximum strength of upper and lower limbs using the number of repetitions [18]. All participants were familiarized with the maximum dynamic load tests on the inclined bench press and leg press, twice in 2 days, before performing the initial and final tests, respecting a rest period of 48 h. The familiarization consisted of the participants performing the maximum dynamic load tests in the inclined bench press (10 kg weight of the bar used) and the leg press (35 kg machine weight) to learn the movement.

### 2.3. Cognitive, Behavioral, and Psychosocial Assessment

The following instruments were used: Short-Form Health Survey—36, to assess the quality of life [19]; Food Markers Questionnaire, for the quality of food intake [20]; the Montreal Cognitive Assessment (MoCA) [21], to assess participants’ cognitive performance; Geriatric Depression Scale and the Baecke Anxiety Scale [22], for the assessment of mood disorder symptoms; Modified Baeke Questionnaire for the Elderly (MBQE) [14], for subjective assessment of the level of physical activity; a triaxial accelerometer (GT3X-BT from ActiGraph) for objective assessment of physical activity level (measuring daily mean, METs, sedentary activity, light activity, moderate activity, and vigorous activity).

Participants used the ActiGraph GT3X-BT accelerometer (Pensacola, Florida, USA) for seven consecutive days, using only 4 days (three weekdays and one weekend day) for statistical calculations, following the recommendations stipulated by Freedson et al. [23].

### 2.4. Biochemical Assay

After a 12 h fasting period, 30 mL of peripheral blood was collected to analyze blood glucose and lipid profile (total cholesterol, HDL cholesterol, LDL cholesterol, and triglycerides). The enzymatic and colorimetric methods were used to analyze total cholesterol (TC) and HDL-cholesterol (HDL), respectively. Fasting blood glucose was measured using the enzymatic system to determine blood glucose (liquefied glucose), following the recommendations in the manufacturer’s manual. To calculate LDL cholesterol (LDL), we used the Friedewald equation [24].

### 2.5. Multicomponent Training

The MT program was carried out for 14 weeks, twice a week, on nonconsecutive days, and each session lasted 90 min, totaling 180 min per week. All sessions were conducted by a physical education professional.

The sessions were divided into four parts:

1. Warmup, including dynamic stretching, coordination, and/or balance exercises,
2. Strength exercises, performed in circuit form, using rubber bands, free weights, shin pads, and body weight,
3. Aerobic endurance and recreational activities (dancing or games),
4. “Cool down”, with relaxation exercises, massage, and stretching [7,9].

The progression of the participants’ load was performed in the week’s first training session. The participants were individually instructed to increase the load in all exercises, always maintaining a moderate/intense intensity on the perceived exertion scale. The physical education professional prepared the classes with different types following a degree of complexity evolution as the participants gain resistance in training. We controlled the training intensity through the Borg Scale, with the objective of perceived exertion at values of 6 and 7 on a scale from 0 to 10, representing a moderate intensity of physical exercise. In addition, a heart rate control system was used for this analysis (Polar Team2, Finland, Brazil, Ribeirão Preto) to ensure reliable use of the Borg scale.

### 2.6. Combined Training

The CT was performed three times a week, lasting 14 weeks, on alternate days, with a total duration of 60 min per session, totaling 180 min per week, and including approximately 30 min of resistance exercise and 30 min of aerobic exercise per session. Aerobic exercise was performed on a treadmill or stationary bicycle. The exercises used in strength training were inclined bench press, table flexor, leg extension, 45° leg press, back pull, articulated row, alternate curl, and cable triceps. Regarding aerobic training, heart rate was continuously monitored through the Polar Team program^2^, which allows simultaneous cardiac monitoring of more than 20 people. During the initial 2 weeks, strength training was performed with 15 to 17 repetitions maximum and aerobic training at 50% of heart rate reserve, while the remainder of the intervention was performed with 10 to 12 repetitions maximum (30 min of strength) to 70% of the heart rate reserve (30 min of training on a treadmill or an ergonomic bike) [13,25]. The load progression was performed individually. Every first day of the training week, the participant was encouraged to increase the weight used in the equipment to perform the number of repetitions instructed by the teacher. Progression in speed or ramp on the treadmill was increased weekly, using the perceived exertion scale and the heart rate monitor to perform progression on the treadmill.

### 2.7. Training Load

We evaluated the intensity of both training modalities using the Subjective Perception of Effort Scale [26], and heart rate during training was assessed using the Polar Team^®^ heart rate meter.

As a method of load quantification, TRIMP (training impulse) was used in both training sessions. TRIMP was calculated on the basis of the subjective perception of effort (SPE) from 0 to 10 during the training period and the total duration of the session expressed in minutes [27]. Monotony was calculated by dividing the average load of the training session during the 14 weeks of training by its standard deviation. The strain was calculated by multiplying monotony and total weekly load [28].

### 2.8. Statistical Analysis

We analyzed the database using the statistical program STATISTICA version 7.0 (TIBCO Software Inc., Hamburg, Germany) and presented the mean and standard deviation. The Shapiro–Wilk test was used to analyze data normality, and we also used the Levene test to assess the equality of variance between the groups. As the analysis showed that the data met the assumptions, ANOVA was used for repeated measures, considering *p* < 0.05%. We adopted Fisher’s post hoc test because it compares all pairs of means and controls the error rate at the significance level. Effect sizes within the group (ES) were calculated as Cohen’s d [29] and classified according to the scale proposed by Rhea [27]. For data for which only the means of the two groups were compared, such as TRIMP, monotony, and deformation, the Student’s *t*-test was adopted, with *p* < 0.05%. Data are presented as mean and standard deviation.

## 3. Results

Age (years), height (cm), body mass (BM) (kg), body mass index (BMI) (kg/m^2^), waist circumference (CM) (cm), hip circumference (cm), and systolic (SBP) and diastolic blood pressure (DBP) (mmHg) are presented in Table 1. The mean age per group was 61.4 ± 4.3 years for CT and 64.1 ± 6.4 years for MT (Table 1). There was no statistical difference between the groups for variables presented in this table.

### 3.1. Upper- and Lower-Limb Maximum Strength, and 6 min Walk Test

We observed the effect of time on the following variables: functional strength of lower limbs by the sit and stand test (F = 60.312; *p* = 0.001), flexibility in the upper limbs by the hand on back test (F = 23.408; *p* = 0.001) and lower limbs by the sit and reach test (F = 6.681; *p* = 0.011), and cardiorespiratory capacity by the 6 min walk test (F = 26.848; *p* = 0.001), as well as strength variables by means of the maximum dynamic repetition test in the upper limbs—incline bench press (F = 10.356; *p* = 0.001) and lower limbs—leg press 45º (F = 13.555; *p* = 0.001).

We observed a group × time interaction for upper-limb strength parameters through the elbow flexion and extension test (F = 4.499; *p* = 0.037). The MT group improved compared to the CT group; for the agility variable (F = 26.598; *p* = 0.001), unlike the functional strength of upper limbs, the group CT demonstrated a better result when compared to MT after 14 weeks of training (Table 2).

### 3.2. Behavioral, Cognitive, and Psychosocial Assessment

We observed the effect of time for the cognitive assessment (MOCA) (F = 15.235; *p* = 0.001) and evaluation of depressive symptoms (GDS) (F = 5.079; *p* = 0.028). There were no significant differences in BAI, MBQE, SF-36, and food intake (Table 3).

### 3.3. Training Load and Physical Activity Level

Regarding the level of physical activity observed by the accelerometer data, we found an effect of time for daily mean (F = 29.058; *p* = 0.001), metabolic equivalent of the task (F = 60.806; *p* = 0.001), sedentary activity (F = 11.818; *p* = 0.001), light activity (F = 16.723; *p* = 0.001), moderate activity (F = 32.794; *p* = 0.001), and vigorous activity (F = 33.115; *p* = 0.001). There were no significant differences in the training load variables (Table 4).

### 3.4. Cholesterol, HDL-c, LDL-c, Triglycerides, and Glycemia

For blood analysis data, a group × time interaction was observed for HDL (F = 5.451; *p* = 0.022); the MT group showed an improvement compared to CT. No other statistical differences were observed (Table 5).

## 4. Discussion

On the basis of the TRIMP, monotony, and strain, it is possible to infer that any differences between the groups in the evaluated parameters were not due to differences between the training since these parameters were not significantly different between the CT and MT groups.

The present study sought to investigate which physical or biochemical benefits or those measured by health evaluation could be provided by combined training compared with multicomponent training in physically inactive middle-aged and older women. We observed significant differences in the comparisons of physical tests. At the same time, there was no difference in the group vs. time interaction for variables included in the cognitive, behavioral, and psychosocial assessment and biochemical assessment.

Our data showed group vs. time difference for elbow flexion and extension and HDL variables for the MT group. In relation to the group vs. time interaction for the CT group, it was observed for both variables that evaluated the maximum repetition of the upper and lower limbs and agility.

The literature already presents several articles claiming that strength should be seen as a fundamental component for improving the performance of other physical abilities and functional performance [30,31,32,33,34].

The different manifestations of force (fast force and resistance force) are influenced by the base component, the maximum force, which is the ability of the neuromuscular system to produce force against an immovable resistance, regardless of the time factor [30,31]. For this reason, the development of maximal strength contributes to improving functional strength, allowing users the ability to transfer maximal strength to everyday tasks in functional strength. In addition to this factor, maximal strength contributes to increased muscle hypertrophy and more remarkable metabolic and morphological adaptations. While functional strength is proven to improve strength and balance, it also helps to enhance joint control due to the greater recruitment of stabilizing muscles in the muscle’s day-to-day tasks [35].

A study by Villareal et al. [36] analyzed participants aged ≥65 years and compared combined training with traditional strength and aerobic training for weight reduction. The training protocol was carried out for 26 weeks, and one of the variables analyzed was muscle strength, assessed using maximum repetition tests. The results showed that the combined training group demonstrated more significant results in relation to the increase in muscle strength compared to the other two training groups, which corroborates our results [36]. Our results demonstrate the increase in strength in a short period of training when compared to the 26 weeks in the study by Villareal et al. [36].

Another study by Johnen and Schott [37], with 45 participants over 65 years, demonstrated that 12 weeks of training were already enough to present improvements in the adopted strength tests. Our population differs from the group presented by Villareal et al. [36] since the participants recruited were older adults diagnosed with frailty. In contrast, our study group comprised middle-aged and older women in good health.

MT stands out for using more than three physical abilities in the same exercise session [7,33]. It may help explain the difference in strength increases between the groups, whereby the CT group demonstrated increased maximum strength, as measured by dynamic maximum load tests. On the other hand, the MT group presented increased functional strength, used in everyday life and measured by the sit and stand up from a chair and elbow flexion and extension tests, both from the Rikli and Jones protocol [16].

The participants maintained acceptable scores according to the recommendations of the Geriatric Depression Scale and Baecke Anxiety Instrument—short version before and after 14 weeks, demonstrating that both training sessions could stabilize anxiety and depression levels. For these and other study variables associated with mental/cognitive diseases, the paradigm that physical training only generates improvement when values are not within the normal range seems to be present [38].

Furthermore, an intervention to generate maintenance of values within normality is essential for healthcare [39,40]. A group × time interaction in the cognitive outcome was not found. The groups both changed after 14 weeks of training. Possibly, the participants were affected by the learning effect since MoCA was applied in fewer than 6 months [41].

To understand their influence on symptoms, a study by LeBouthillier and Asmundson [42] investigated the effect of aerobic and strength training on anxiety in 48 individuals pre-diagnosed with anxiety and aged between 18 and 65. The training protocol lasted 4 weeks, and assessments took place at the beginning, weekly, and 1 month after the end of training. The resulting data demonstrated that physical training provided anxiety reductions for both participants assigned to the aerobic training group and those assigned to strength training. Another study by Rogers et al. [43] with 222 middle-aged women who were postoperative for breast cancer investigated the effect of MT in reducing fatigue, anxiety, and depression.

The training protocol was performed for 3 months and showed significant reductions in symptoms of depression and anxiety, but these findings were not reflected in our data. In the studies presented, participants were diagnosed with symptoms of depression and anxiety, with initial values different from our participants, who were not diagnosed with any symptoms of depression and anxiety [40,41]. Together, these findings corroborate the trainability principle explained in the previous paragraph; worse initial values correlated with a greater potential for training to generate benefits.

For biochemical data, our findings showed a significant difference for HDL; the MT group presented an increase in HDL levels after 14 weeks of training compared to the CT group [44]. The literature shows that a 1% increase in HDL levels is associated with a 3% reduction in cardiovascular mortality rates [45,46]. A study by Heubel et al. [47] investigated the influence of multicomponent training on physical capacity and diabetes mellitus in 13 women with a mean age of 68 ± 6 years. The authors obtained significant results after 16 weeks of training in the participant’s fasting blood glucose and HDL profiles, who presented outliers of normality standards [47]. These data corroborate our results regarding HDL.

The MT is described by the combination of resistance exercises, strength, balance, and flexibility, in addition to relying on aerobic metabolism [47]. As the elderly become trained, changes in blood lipids are caused by regular exercises, such as increased lipoprotein lipase activity in skeletal muscle, in addition to promoting increased capillary density and potential in the removal and use of fatty acids [48,49]. This may explain the changes in the increase in HDL levels for the MT group. Notably, these findings of the increase in HDL levels observed for the MT group were not presented for the CT group.

The current study brings two novelties to the scientific community related to physical exercise: (1) using physically inactive women aged 50 to 70 years to compare two training protocols, CT, involving weight training plus cardiorespiratory capacity, and MT, combining training with different abilities in one single session; (2) maintaining the training volume of both at 180 min and ensuring similar intensities to eliminate differences in stimuli between the different training protocols.

Our study had some limitations. Firstly, we did not use the standard gold test to measure cardiorespiratory capacity, as this variable could have affected the results of the comparison between training sessions. Secondly, we did not use a control group. However, the 6 min walk test has been validated and widely used in the context of aging. It is important to note that the aim was not to see the effect of physical exercise but to compare two different intervention methods for women aged 50 to 70 years.

## 5. Conclusions

Both training sessions showed significant improvements in physically inactive middle-aged and older women after 14 weeks of training, corroborating the study’s initial hypothesis. When we looked at which training led to better results, combined training showed more significant results than 14 weeks of multicomponent training. It is worth mentioning that each type of training improved different types of strength in each group. The CT group showed an improvement in the maximum strength and agility tests, while the MT showed better functional strength and HDL results.

## Figures and Tables

**Table 1:** Mean ± standard deviation of age, anthropometric measurements, and blood pressure pre- and post-intervention (MT n = 32; CT n = 39).

Variables	Group	Pre	Post	*p* (Group)	*p* (Time)	*p* (Group × Time)
Age (years)	CT (n = 39)	61.4 ± 4.3		-	-	-
	MT (n = 32)	64.2 ± 6.4				
Height (m)	CT (n = 39)	1.56 ± 0.06		-	-	-
	MT (n = 32)	1.55 ± 0.04				
BM (kg)	CT (n = 39)	74.7 ± 8.9	75.3 ± 7.7	0.071	0.847	0.360
	MT (n = 32)	69.9 ± 9.9	69.5 ± 10.1			
BMI (kg/m^2^)	CT (n = 39)	30.5 ± 2.8	30.4 ± 2.4	0.062	0.501	0.886
	MT (n = 32)	28.6 ± 3.4	28.5 ± 3.8			
WC (cm)	CT (n = 39)	97.2 ± 7.3	96.3 ± 8.3	0.939	0.842	0.344
	MT (n = 32)	96.2 ± 9.0	96.9 ± 7.8			
HC (cm)	CT (n = 39)	108.5 ± 7.2	106.4 ± 7.5	0.188	0.095	0.428
	MT (n = 32)	105.0 ± 7.8	104.3 ± 8.0			
SBP (mmHg)	CT (n = 39)	131.6 ± 11.9	125.7 ± 11.5	0.629	0.410	0.113
	MT (n = 32)	126.2 ± 16.7	128.1 ± 10.0			
DBP (mmHg)	CT (n = 39)	77.8 ± 6.97	76.3 ± 6.6	0.636	0.472	0.698
	MT (n = 32)	76.4 ± 9.87	75.9 ± 8.4			

Note: BM: body mass; BMI: body mass index; WC: waist circumference; HC: hip circumference; SBP: systolic blood pressure; DBP: diastolic blood pressure; CT: combined training; MT: multicomponent training. Repeated-measures two-way ANOVA was used, followed by the Fisher LSD post hoc test.

**Table 2:** Mean ± standard deviation of the tests used to assess pre- and post-intervention physical abilities.

Variables	Group	Pre	Post	*p* (Group)	*p* (Time)	*p* (Group × Time)	Effect Size (Cohen’s d)
EFE (reps)	CT (n = 39)	16.7 ± 3.0	21.1 ± 3.1	0.022 *	0.001 *	0.037 *	1.83
	MT (n = 32)	13.6 ± 2.7	20.7 ± 4.1 ^†^				2.49
SAS (reps)	CT (n = 39)	13.5 ± 3.4	18.1 ± 4.1	0.574	0.001 *	0.342	1.57
	MT (n = 32)	12.4 ± 2.7	18.2 ± 4.2				2.13
RM LL (kg)	CT (n = 39)	126.5 ± 24.4	216.4 ± 29.4 ^†^	0.027 *	0.001 *	0.002 *	3.99
	MT (n = 32)	125.2 ± 19.4	157.5 ± 15.9				2.29
RM UL (kg)	CT (n = 39)	20.2 ± 4.1	35.9 ± 4.7 ^†^	0.001 *	0.001 *	0.001 *	4.25
	MT (n = 32)	17.4 ± 2.6	21.4 ± 3.5				1.71
HOB (cm)	CT (n = 39)	−8.9 ± 7.3	−5.3 ± 6.5	0.541	0.001 *	0.139	0.84
	MT (n = 32)	-6.8 ± 8.6	-4.8 ± 7.5				0.60
SAR (cm)	CT (n = 39)	0.4 ± 5.8	3.0 ± 5.9	0.475	0.011 *	0.445	0.76
	MT (n = 32)	−0.1 ± 5.6	1.2 ± 5.8				0.58
Walk (m)	CT (n = 39)	523 ± 66	579 ± 53	0.664	0.001 *	0.853	1.28
	MT (n = 32)	531 ± 47	583 ± 71				1.25
Agility (s)	CT (n = 39)	27.1 ± 3.9	22.5 ± 3.0 ^†^	0.953	0.001 *	0.001 *	−0.83
	MT (n = 32)	25.1 ± 2.7	24.4 ± 2.8				0.12

Note: EFE: elbow flexion and extension; SAS: sit and stand; RM: maximum repetition; LL: lower limbs; UL: upper limbs; HOB: hands on back; SAR: sit and reach; reps: repetitions; CT: combined training; MT: multicomponent training; <0.35: trivial; 0.35 to 0.80: small effect; 0.80 to 1.50: moderate effect; ≥1.50: large effect for effect size Cohen’s d. * Statistical difference *p* < 0.05 for pre versus post; ^†^ group×time difference. Repeated-measures two-way ANOVA was used, followed by the Fisher LSD post hoc test.

**Table 3:** Mean ± standard deviation of global health assessment pre- and post-intervention.

Variables	Group	Pre	Post	*p* (Group)	*p* (Time)	*p* (Group × Time)	Effect Size (Cohen’s d)
MOCA (points)	CT (n = 26)	21.9 ± 3.6	24.5 ± 2.6	0.547	0.000 *	0.256	1.24
	MT (n = 26)	22.0 ± 3.1	23.3 ± 2.8				0.83
GDS (points)	CT (n = 26)	3.3 ± 3.2	3.0 ± 2.7	0.908	0.028 *	0.403	0.30
	MT (n = 26)	3.4 ± 2.5	2.6 ± 2.2				0.09
BAI (points)	CT (n = 26)	4.4 ± 4.8	4.8 ± 4.6	0.474	0.661	0.802	0.48
	MT (n = 26)	3.4 ± 2.8	3.6 ± 3.1				0.46
MBQE (points)	CT (n = 26)	4.6 ± 3.1	6.5 ± 3.5	0.013	0.026	0.620	0.96
	MT (n = 26)	7.3 ± 4.3	8.5 ± 3.5				0.69
SF-36 DM (points)	CT (n = 26)	57.2 ± 5.7	56.5 ± 9.3	0.357	0.731	0.464	0.31
	MT (n = 26)	53.8 ± 6.3	55.9 ± 7.6				0.69
SF-36 DF (points)	CT (n = 26)	66.1 ± 4.3	64.3 ± 5.4	0.702	0.770	0.135	0.07
	MT (n = 26)	63.9 ± 5.9	65.1 ± 7.1				0.57
Food intake (points)	CT (n = 26)	18.7 ± 8.1	22.0 ± 7.4	0.306	0.193	0.065	0.81
	MT (n = 26)	17.9 ± 7.6	17.3 ± 8.4				0.33

Note: MOCA: Montreal Cognitive Assessment; GDS: Geriatric Depression Scale; BAI: Baecke Anxiety Instrument; MBQE: Modified Baecke Questionnaire for Older Adults; SF36: quality-of-life questionnaire; PD: physical domain; MD: mental domain. CT: combined training; MT: multicomponent training; <0.35: trivial; 0.35 to <0.80: small effect; 0.80 to 1.50: moderate effect; ≥1.50: large effect for effect size Cohen’s d. * Statistical difference *p* < 0.05 for pre vs. post. Repeated-measures two-way ANOVA was used.

**Table 4:** Mean ± standard deviation of the values of training intensities and physical activity level pre- and post- intervention.

Variables	Group	Pre	Post	*p* (Group)	*p* (Time)	*p* (Group × Time)	Effect Size (Cohen’s d)
TRIMP (a.u.)	CT (n = 36)	630 ± 48		0.109			
	MT (n = 25)	675 ± 37					
Monotony (a.u.)	CT (n = 36)	4.6 ± 1.3		0.630			
	MT (n = 25)	4.5 ± 1.2					
Strain (a.u.)	CT (n = 36)	829 ± 236		0.500			
	MT (n = 25)	806 ± 221					
Daily average (counts)	CT (n = 36)	33.5 ± 25.3	150.6 ± 121.2	0.565	0.000 *	0.398	2.01
	MT (n = 25)	34.9 ± 28.2	120.1 ± 96.8				1.82
METs (counts)	CT (n = 36)	1.0 ± 0.0	2.1 ± 0.9	0.973	0.000 *	0.986	2.97
	MT (n = 25)	1.0 ± 0.0	2.1 ± 1.0				2.77
Sedentary activity (counts)	CT (n = 36)	5964 ± 887	16236 ± 11534	0.484	0.001 *	0.519	2.08
	MT (n = 25)	6148 ± 936	21197 ± 19646				1.93
Light activity (counts)	CT (n = 36)	2471 ± 495	4287 ± 2505	0.253	0.000 *	0.379	1.58
	MT (n = 25)	2287 ± 485	3456 ± 2200				1.28
Moderate activity (counts)	CT (n = 36)	72.6 ± 68.0	232.8 ± 177.8	0.294	0.000 *	0.134	1.68
	MT (n = 25)	70.5 ± 67.7	163.5 ± 114.3				1.44
Vigorous activity (counts)	CT (n = 36)	0.1 ± 0.1	3.9 ± 3.2	0.304	0.000 *	0.196	2.81
	MT (n = 25)	0.2 ± 0.4	2.6 ± 2.4				2.21

Note: METs: metabolic equivalents of task; CT: combined training; MT: multicomponent training; <0.35: trivial; 0.35 to 0.80: small effect; 0.80 to 1.50: moderate effect; ≥1.50: large effect for effect size Cohen’s d. * Statistical difference *p* < 0.05 for pre versus post. Repeated-measures two-way ANOVA was used.

**Table 5:** Mean ± standard deviation of biochemical blood analyses pre- and post-intervention.

Variables	Group	Pre	Post	*p* (Group)	*p* (Time)	*p* (Group × Time)	Effect Size (Cohen’s d)
Cholesterol (mg/dL)	CT (n = 37)	210.8 ± 22.6	204.4 ± 28.2	0.308	0.862	0.055	0.10
	MT (n = 25)	194.4 ± 31.2	202.2 ± 34.9				0.63
HDL-c (mg/dL)	CT (n = 37)	58.6 ± 11.5	56.8 ± 12.5	0.081	0.564	0.022 *	0.19
	MT (n = 25)	49.6 ± 10.0	52.6 ± 12.1 ^†^				0.67
LDL-c (mg/dL)	CT (n = 37)	124.6 ± 21.6	121.4 ± 24.9	0.819	0.916	0.378	0.20
	MT (n = 25)	119.9 ± 27.6	122.4 ± 28.7				0.49
Triglycerides (mg/dL)	CT (n = 37)	138.1 ± 69.3	130.8 ± 63.1	0.814	0.760	0.148	0.23
	MT (n = 25)	124.6 ± 60.7	135.9 ± 97.8				0.54
Glycemia (mg/dL)	CT (n = 37)	107.2 ± 17.5	104.0 ± 14.0	0.164	0.112	0.731	0.14
	MT (n = 25)	100.3 ± 8.8	98.2 ± 9.8				0.20

Note: HDL-c: high-density lipoprotein; LDL-c: low-density lipoprotein; CT: combined training; MT: multicomponent training; <0.35: trivial; 0.35 to 0.80: small effect; 0.80 to 1.50: moderate effect; ≥1.50: large effect for effect size Cohen’s d. * Statistical difference *p* < 0.05 for pre versus post; † group × time difference. Repeated-measures two-way ANOVA was used, followed by the Fisher LSD post hoc test.

## Data Availability

The data presented in this study are available on request from the corresponding author. The data are not publicly available due to consent provided by participants.
